# Whole genome sequencing of a *Domibacillus* strain from the Cuatro Ciénegas basin that tends to act as an altruist

**DOI:** 10.1128/mra.00230-24

**Published:** 2024-10-21

**Authors:** Diego Aguilera-Najera, Ulises E. Rodriguez-Cruz, Brenda Vazquez, Christian Fernandez, Eunice Martinez-Perez, Rosalinda Tapia-Lopez, Valeria Souza

**Affiliations:** 1 Ecology Evolutionary Department, Institute of Ecology, National Autonomous University of Mexico, Mexico City, Mexico; 2 National School of Higher Education Juriquilla Unit, National Autonomous University of Mexico, Mexico City, Querétaro, Mexico; The University of Arizona, Tucson, Arizona, USA

**Keywords:** Cuatro Ciénegas, genome, *Domibacillus*

## Abstract

*Domibacillus* sp*. 8LH* is a whitish bacterium isolated from the pools of the Cuatro Cienegas Basin (CCB) in the state of Coahuila and belongs to the *Bacillaceae* family. It grows in circular colonies of about 6 mm in diameter and is capable of forming biofilms. This strain was identified because, in previous experiments in our laboratory, it presented altruistic interactions when co-cultured with bacteria of the genus *Bacillus* that participate in the nitrogen cycle. This altruistic behavior confers to this *Domibacillus* strain a potential use for the construction of bacterial consortia with diverse biotechnological applications.

## ANNOUNCEMENT

During the experimental stage of the project, we studied the interactions of bacteria from two different sites. We found an interesting phenotype present in one of the bacteria, “Strain 8.” During the experimental phase, “strain 8” helped the growth and development of different bacteria involved in the nitrogen cycle (Martinez-Perez,E. , Zaragoza-Fernandez,A. , Marquez-Cianci, L. , Rosas-Barrera,M. , Aguilera-Najera,D. ,Tapia-Lopez,R. ,Rodriguez-Cruz,U.E. , Souza-Saldivar,V. and Eguiarte,L.E. unpublished data). This phenotype was present regardless of the origin of the bacteria, the Cuenca de Cuatro Cienegas (CCB), or two mangrove sites in Topolobambo, Sinaloa. This bacterial strain was extracted from the lagoon of Los Hundidos within CCB; an oasis located in the state of Coahuila, Mexico.

The evidence obtained in this work indicates that the sequenced strain is within the phylum *Bacillota,* class *Bacilli,* order *Bacilliales*, family *Bacillaceae,* and belongs to the genus *Domibacillus*. This genus is composed of nine species at the time of writing. The strain *Domibacillus* sp. *LH8* has unique characteristics described so far in the genus, presenting a cooperative behavior in the presence of *Bacillaceae* strains that possess genes within the nitrogen cycle.

## MATERIALS AND METHODS

Water samples were taken from the Los Hundidos lagoon (geographic coordinates 26° 52' 11.84" N; 102° 01' 12.60" W), located in the Pozas Rojas system in CCB. Surface water samples were collected in sterilized bottles and kept in a cold cooler until use.

In the laboratory, the water was filtered on sterile GF/F filters (0.2 mm nominal pore size; Whatman) using a Millipore filtration device. Each GF/F filter was introduced into marine media plates (Difco & BBL/BD Diagnostics, 1984), and incubated at 30°C for 3 days. After achieving independent colony growth, they were inoculated individually on the same media under similar conditions for 1 day. After morphological and phenotypical characterization of a single colony of strain, 8LH was grown in liquid medium, one portion of this culture was used for glycerol storage at −70°C and the other portion was used for glycerol storage at −70°C. Samples were collected under SEMARNAT scientific permit SGPA/DGVS/04225/21.

DNA was extracted from an axenic culture with the DNAeasy Blood & Tissue QIAGEN’s DNA Library extraction kit (according to the manufacturer instructions), DNA quality was checked by electrophoresis, and libraries were prepared with the Illumina DNA LIBRARY PREP 2, the DNA fragments were done by tagmentation. Libraries of 600 bp were sequenced using the Illumina Miseq platform with 2 × 150 bp paired libraries using MiSeq Reagents Kit v2. A total of 2,714,126 reads paired-end were obtained. We performed a quality analysis of the raw reads using the FASTQC program (1). To perform the *de novo* assembly we used three different software tools; VELVET (2), SPADES (3), and MEGAHIT (4) in order to compare the obtained results and select the best one. The comparison was made using the QUAST (5) bioinformatic program. Finally, The genome was annotated using PROKKA (6).

**Fig 1:**
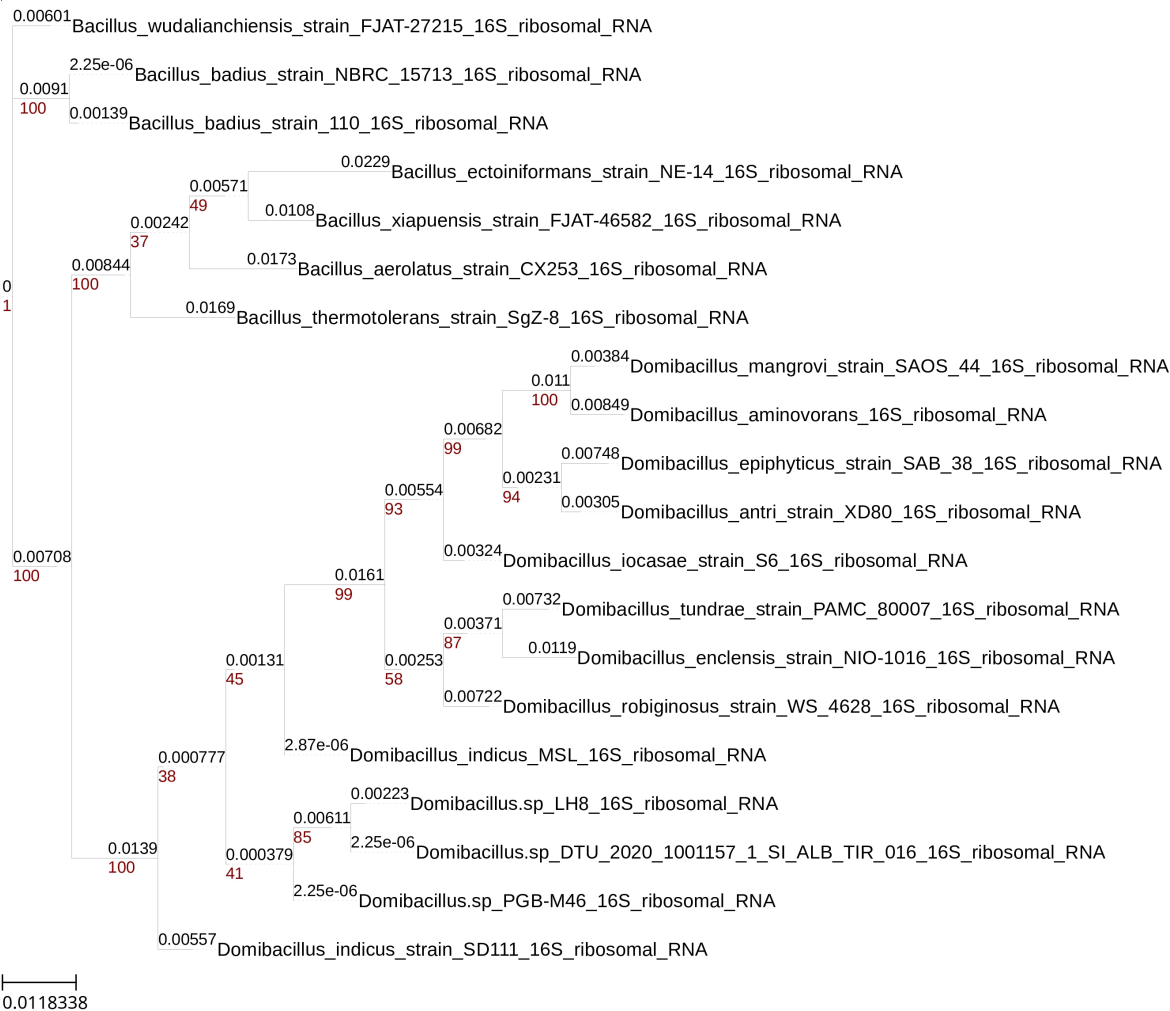
Phylogenetic tree constructed with IQ-Tree Phylogenetic tree inferred with the maximum likelihood method using IQ-Tree. The 16S sequences of organisms of the genera Domibacillus and Bacillus.(13–20).

The sequences of the 16S rRNA subunit, previously obtained with Barrnap software, were used for taxonomic identification. The sequences were compared with sequences from the NCBI, SILVA(7), and RDP databases using Blast. Next, the closest sequences were aligned using MUSCLE (8). The result of this alignment was used to infer a maximum likelihood phylogenetic tree by nucleotide substitution model selection using the IQ-TREE (9, 10) web service (available at http://www.iqtree.org/). As an additional method, to improve the accuracy of our results, we used the tools ANIclustermap and FastANI (Yoshiyama Y, 2022; 11, 12), to measure the ANI of our genome with the rest of the genomes of the genus used with the phylogenetic tree (Fig. 1).

## RESULTS AND DISCUSSION

The best assembly obtained was obtained with SPADES. The final genome has a final length of approximately 5.0 MB, an average GC content of 47% whole genome, and an N50 of 137.12 kb consisting of nine scaffolds whose coverage is above 35 and 4,710 genes according to the results reported by Prokka. Using data obtained from 16S rRNA sequences and SILVA and RDP data, we found matches between different species of the genera *Domibacillus* and *Bacillus*. To confirm our results, we used the fastANI program. According to fastANI the new strain shares 95.8% nucleotide identity with Domibacillus sp. DTU, coinciding with results obtained with 16S sequences.

This work contributes to the understanding of the taxonomic classification of bacteria, adds to the growing body of literature on the genus *Domibacillus*, and expands our knowledge of the diversity and distribution of this group of bacteria.

### Software

All the programs used in this methodology were performed using the default parameters unless otherwise stated in the methodology.

- Fastqc (version 0.12.0)
- VELVET (version 1.2.10 )
- SPADES (version 3.15.5 )
- MEGAHIT (version 1.2.9 )
- QUAST (version 5.2 )
- MUSCLE (version 3.8.425 )
- Barrnap (version 3 )
- FastANI (version 1.33)
- ANIclustermap (version 1.3 )
- IQ-TREE (version 1.6.12)
- PROKKA (version 1.14.5)
- PGAP (version 6.6 )

## Data Availability

Complementary images of the results presented in this work are available at Figshare DOI:https://doi.org/10.6084/m9.figshare.25371538.v2.(24–30) Both raw reads and assembled data were submitted on the NCBI platform to the following Bioproject: PRJNA1013342. The genome assembly is registered with the ID: JAVJNG000000000. The raw read data are registered in the SRA: SRR25930181. The public version of the genome was annotated using PGAP (21–23), while this work was performed using a Prokka annotation.
